# 超声提取-高温燃烧吸收-离子色谱法测定纺织品中可吸附有机卤化物

**DOI:** 10.3724/SP.J.1123.2021.03018

**Published:** 2022-01-08

**Authors:** Youchao DING, Lihua CAO, Liping ZHOU, Kai QIAN, Juan TANG, Jia ZHOU, Shaowei DONG

**Affiliations:** 1.南京海关工业产品检测中心, 江苏 南京 210019; 1. Nanjing Customs Industrial Products Testing Center, Nanjing 210019, China; 2.南昌海关, 江西 南昌 330009; 2. Nanchang Customs,Nanchang 330009, China; 3.南京金检检验有限公司, 江苏 南京 210019; 3. Nanjing Jinjian Inspection Co., Ltd, Nanjing 210019, China

**Keywords:** 离子色谱, 超声提取, 高温燃烧吸收, 可吸附有机卤化物, 纺织品, ion chromatography (IC), ultrasonic extraction, high temperature combustion absorption, adsorbable organic halogens (AOX), textiles

## Abstract

建立了一种对纺织品中可吸附有机卤化物(AOX)的超声提取-高温燃烧吸收-离子色谱定量检测分析新方法。该方法采用超声方式提取纺织品中的AOX,提取液加入活性炭进行振荡吸附,并用酸性硝酸钠溶液对无机卤化物进行去除。采用程序升温的氧化燃烧方式对吸附AOX的活性炭进行裂解、燃烧及气化,其产生的卤化氢等气体随载气进入吸收液并完全转化为无机卤素阴离子,采用离子色谱分离测定,外标法定量。实验优化了超声提取时间、活性炭用量、燃烧气及其流量、燃烧升温程序、吸收液和吸收方式等前处理条件,并对离子色谱的仪器分析条件如色谱柱、柱温及淋洗液流速等进行优化。结果表明,氟、氯、溴、碘4种卤素离子的标准溶液在0.02~10 mg/L范围内呈线性关系,线性相关系数(*R*^2^)均在0.999以上;AOX测定的方法定量限为0.10~0.50 mg/kg。以棉、毛和涤纶3种不同种类的阴性纺织样品作为样品基质,选取典型的有机卤化物进行加标,在低、中、高3个加标水平下测得AOX的平均回收率为82.3%~98.7%,相对标准偏差(RSD, *n*=7)为2.0%~5.7%,表明方法具有良好的回收率和精密度。将该方法应用于实际纺织样品的测定,检出了不同含量的AOX,且重复性好。研究建立的方法通过采用活性炭的振荡吸附、程序升温的高温氧化燃烧方式和多孔吸收瓶的二级吸收方法,提高了AOX转化为无机卤素的回收率;同时利用离子色谱仪器选择性好、灵敏度高的特点成功地一次性分离检测4种AOX,且无杂质离子的干扰。该方法简单、准确、可靠,满足国内外法规和标准对纺织品中AOX的限量要求,适用于纺织品中AOX的分析测定。

## Abstract (en)

Adsorbable organic halogens (AOX) are often introduced or produced in textile and dyeing processes, such as the chlorination shrink proof process of wool, the bleaching process by sodium hypochlorite and chlorite, the dry-cleaning process by chlorinated solvent, etc. However, part of AOX is difficult to biodegrade and is a persistent bioaccumulative toxic substance with high fat solubility. To promote clean production of textiles and to protect the health of consumers, a conventional method for the detection of AOX in textiles must be established urgently. In this study, a new method was developed for the determination of AOX in textiles by ultrasonic extraction-high temperature combustion absorption-ion chromatography (IC). In this method, AOX in textiles were extracted by ultrasonic extraction at room temperature with ultra-pure water as solvent. Activated carbon was added to the extraction solution for oscillatory adsorption and removal of inorganic halides with acidic sodium nitrate solution. The AOX adsorbed on activated carbon were cracked, burned, and gasified by the oxidative combustion method with a programmed heating mode. The product hydrogen halide gas entered the absorption solution with the carrier gas, followed by separation and determination by IC with external standard method of quantification. During the experiment, the pretreatment conditions were optimized, including the extraction time of AOX, the amount of activated carbon, the combustion gas and its flow rate, the temperature program for high-temperature oxidation combustion, the absorption method, and the absorption solution. The instrument conditions of ion chromatography, including the chromatographic column, column temperature, eluent, and its flow rate, were also optimized. The results showed that the method was linear in the range of 0.02-10 mg/L for the standard solutions of fluorine, chlorine, bromine, and iodine ions, and the correlation coefficients (*R*^2^) were greater than 0.999. The limits of quantification of the method for AOX were 0.10-0.50 mg/kg. The negative textile samples of cotton, wool, and polyester were used as the sample matrix, and typical organic halogens were selected for standard addition and recovery. At low, medium, and high spiked levels, the average recoveries of AOX in cotton, wool, and polyester fiber were 82.3%-98.7%. The corresponding relative standard deviations (RSDs, *n*=7) were 2.0%-5.7%, indicating that the method had good recovery and precision. This method was used to determine actual textile samples, and AOX at different contents were detected in blue coated polyester fabric and black modal fabric with good repeatability. The established method improved the recoveries of AOX converted into inorganic halogens via oscillating adsorption of activated carbon, high-temperature oxidation combustion with a programmed heating mode, and secondary absorption using a porous absorption bottle. Meanwhile, the separation and detection of halogen ions was successfully conducted using the ion chromatography instrument with good selectivity and high sensitivity, without any interference of impurity ions. The method is simple, accurate, and reliable, and fully meets the limit requirements of domestic and foreign regulations and textile standards; the method is also suitable for the detection and analysis of AOX in textiles.

可吸附有机卤化物(AOX)最早于1976年被提出,是指溶解在水中并能被活性炭吸附的一类有机卤化物,包括有机氯化物、有机溴化物和有机碘化物,但不包括有机氟化物^[1,2,3]^。工业生产的有机卤化物常被用作农药、消毒剂、有机溶剂、药物和阻燃剂等,但是部分AOX难以生物降解,是持久性的生物累积性有毒物质,且具有较高的脂溶性,极易积存于人体和动物的脂肪组织内,会对人类和动物造成伤害,部分AOX类物质已被证实具有潜在致癌和致突变性^[4,5,6]^。

纺织和染整工艺过程经常会引入或产生AOX,如羊毛的氯化防缩处理过程、次氯酸钠和亚氯酸盐的漂白过程、氯代溶剂干洗过程等,而AOX在生物体内容易长期残留,且键合能力十分显著,能与人体蛋白质或核酸发生作用,而且极易在生态环境中积聚并通过食物链影响人的健康并造成危害^[7,8,9]^。欧盟指令2002/371/EC规定了人造纤维中AOX的含量不超过250 mg/kg;瑞士的“Coop Nature Line”(自然合作阵线)要求纤维或织物上总AOX低于1 g/kg; 2011版的全球有机纺织品标准(GOTS 3.0)对含氯苯酚和氯化溶剂实施了禁用,同时还规定在生产过程中相关原料的投入量,最终产品中AOX的含量小于5.0 mg/kg^[10,11]^。我国标准《纺织纤维中有毒有害物质的限量》同样对人造纤维中AOX提出不超过250 mg/kg的限量要求^[12]^。

为了满足国内外技术法规和标准对纺织品中AOX的限量要求,促进纺织品清洁生产、保护消费者健康,亟须建立一种用于纺织品中AOX的检测方法。目前,国内外法规和标准推荐的检测方法为《纸浆、纸和纸板总氯和有机氯的测定》^[13]^,但是通过研究发现,该标准无论是在应用领域还是检测对象方面,并不适用于纺织品中AOX的检测,国内外尚未制定相关纺织品中AOX含量的测定标准。当前AOX的研究集中于废水、土壤等环境领域^[14,15,16,17]^,而对于纺织品领域内AOX检测方法的研究很少,沈锦玉等^[18]^提出了振荡提取-离子色谱(IC)测定纺织品中AOX含量的检测方法,但只针对可吸附有机氯(AOCl)。对于纺织品中AOX的定义,陈荣圻^[19,20]^发表了专论,对于含卤纺织品与有机卤化物的概念进行了充分讨论和验证,提出了纺织品过程中使用的含氟卤化物也属于有机卤化物的概念。本文采用了超声提取-高温燃烧吸收-离子色谱的方法实现了同时测定纺织品中可吸附有机氟(AOF)、可吸附有机氯(AOCl)、可吸附有机溴(AOBr)和可吸附有机碘(AOI)的分析新方法,通过采用活性炭振荡方法吸附样品中提取的AOX,高温燃烧的程序升温方式和多孔吸收瓶的二级吸收方法提高了AOX转化为无机卤素的回收率,并通过优化离子色谱的仪器条件,建立了一套完善、准确可靠地应用于纺织品中AOX的测定方法。

## 1 实验部分

### 1.1 仪器、试剂与材料

Metrohm 940离子色谱仪(瑞士Metrohm公司); AOX-3有机卤素燃烧炉(杭州卓驰仪器有限公司); SQP224-1CN分析天平(感量0.01 g和0.0001 g,德国Sartorius公司); Milli-Q超纯水系统(美国Millipore公司); B5510E超声波清洗器(美国Branson公司); HY-4A调速多用振荡器(常州润华电器有限公司);水相针式滤器(13 mm×0.22 μm,上海安谱实验科技股份有限公司)。

氟离子、氯离子、溴离子、碘离子标准溶液(1000 mg/L)购于美国NSI公司;浓硝酸、硝酸钠、无水碳酸钠、碳酸氢钠均为优级纯,购于上海国药集团有限公司;4-氟苯酚(99%)、4-氯苯酚(99%)、4-溴苯酚(98%)、4-碘苯酚(98%)、五氯苯(98%)、六溴环十二烷(95%)购于阿拉丁试剂(上海)有限公司;活性炭:吸附力(碘值)≥1050,杂质(氯化物)≤0.0015%,颗粒度10~50 μm,购于德国Analytikjena公司;聚碳酸酯滤膜:规格47 mm×0.4 μm,购于美国Millipore公司;超纯水:电阻率≥18.2 MΩ·cm;氧气:纯度≥99.9%。

### 1.2 标准溶液的配制

分别准确移取2.00 mL氟离子、氯离子、溴离子、碘离子标准溶液,于100 mL棕色容量瓶中,用超纯水配制成质量浓度为20 mg/L的混合标准储备液,于4 ℃冰箱冷藏保存。使用时用超纯水逐级稀释,配制成0.02、0.05、0.10、0.20、0.50、1.0、2.0、5.0、10.0 mg/L的系列标准溶液。

### 1.3 样品前处理

1.3.1 样品中AOX的提取

将样品剪碎至5 mm×5 mm大小以下,称取2 g(精确至0.01 g)样品,置于具塞锥形瓶中,加入100 mL超纯水,在常温下超声提取60 min。用砂芯漏斗过滤并用25 mL超纯水洗涤试样和锥形瓶,收集所有滤液于另一锥形瓶中,待用。在滤液中加入50 mg活性炭,以150次/min的速率在常温下振荡处理60 min。使用聚碳酸酯滤膜用溶剂抽滤装置抽滤,之后用50 mL酸性硝酸钠溶液(0.85 g/L, pH=3)漂洗活性炭、锥形瓶和过滤器内壁,最后用少量超纯水洗涤,真空抽滤至干,将处理后的活性炭连同滤膜取下,待用。

1.3.2 高温燃烧吸收

在有机卤素燃烧炉的可编程控制面板上设置升温程序:初始温度100 ℃,保持2 min,之后15 min内升至950 ℃,保持5 min,继续通入氧气进行吹脱5 min。调节氧气压力和流量计,使得燃烧管内管氧气流量为100~120 mL/min,外管氧气流量为40~60 mL/min。用约3 cm的硅胶管连接燃烧管出口和多孔吸收瓶入口,用石棉布包裹连接处,防止气体冷凝。

将处理后的活性炭连同滤膜一起放入样品舟中并推入燃烧炉的中间位置,即高温区,入口处用橡胶塞封闭。采用离子色谱淋洗液作为吸收液,两个50 mL的多孔吸收瓶串联进行二级吸收。分别准确移取10.00 mL吸收液,置于两个多孔吸收瓶中,连接燃烧管调节氧气流量,使得吸收瓶中的气泡均匀鼓出。启动升温程序进行高温氧化燃烧,其产生的卤化氢等气体被完全吹出燃烧炉,从而被吸收液吸收。

取下二级吸收瓶,将吸收液定量转移至25 mL容量瓶中,用少许淋洗液继续洗涤吸收瓶并定容至刻度,水相针式滤器过滤,滤液供离子色谱测定。

### 1.4 分析条件

色谱柱:Metrosep A Supp 5-250/4.0型柱(250 mm(长度)×4.0 mm(内径));保护柱:Metrosep A Supp 5 Guard/4.0专用保护柱;淋洗液:3.2 mmol/L Na_2_CO_3_和1.0 mmol/L NaHCO_3_;柱温:35 ℃;柱流速:0.7 mL/min;进样量:20 μL。

## 2 结果与讨论

### 2.1 前处理条件的考察

2.1.1 提取时间的选择

根据AOX的定义^[1,2,3]^,选取超纯水作为提取溶剂;考虑到纺织品纤维与AOX的结合紧密程度,采用超声提取方式在常温下对纺织品中的AOX进行提取。

为了考察超声提取时间的影响,试验选取阴性纯棉样品作为研究对象,向其中添加典型有机卤化物(4-氟苯酚、4-氯苯酚、4-溴苯酚、4-碘苯酚、五氯苯和六溴环十二烷),使样品中各卤素的含量均达到50 mg/kg,之后在超声时间分别为20、40、60、80和100 min的条件下进行加标回收试验,结果见图1。从图中可以看出,超声提取时间对AOX的提取率有较大影响,随着超声提取时间的增加,加标回收率逐渐增大,在60 min时达到稳定并基本保持恒定。因此,选取60 min的提取时间作为最佳提取时间。

**图 1:**
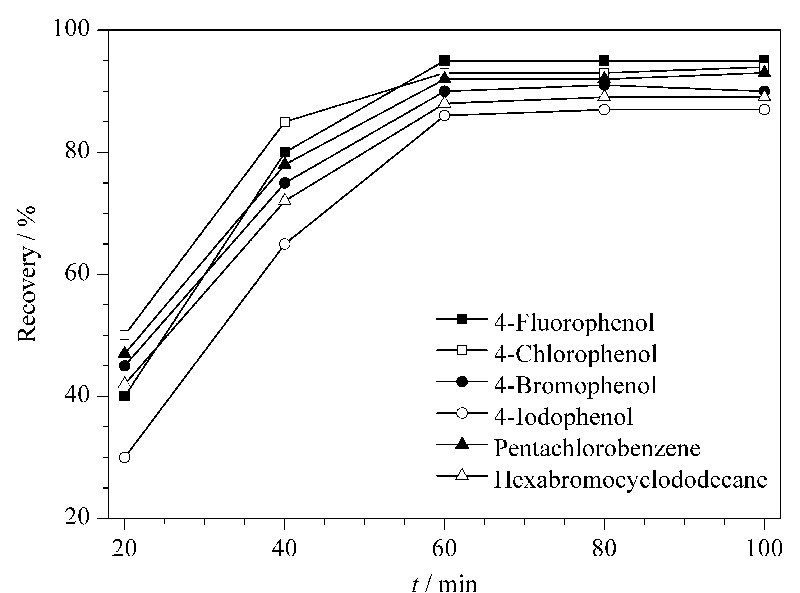
超声提取时间对样品加标回收率的影响

2.1.2 活性炭用量的选择

活性炭作为一种常用的吸附剂能够对有机卤化物进行富集,在活性炭颗粒表面积基本一致的情况下,活性炭用量的多少决定了其吸附效果,因此需要通过试验选取适量的活性炭,以便能够将样品中的AOX完全吸附。考虑到振荡吸附是一个动态平衡过程,选取振荡吸附时间60 min,在活性炭加入量分别为20、50和80 mg的条件下进行阴性加标样品(50 mg/kg)的回收试验(见图2)。结果显示,随着活性炭用量的增加,加标回收率明显提高,活性炭用量在50 mg和80 mg时的结果相差不大,因此选取50 mg的活性炭作为最佳用量。

**图 2:**
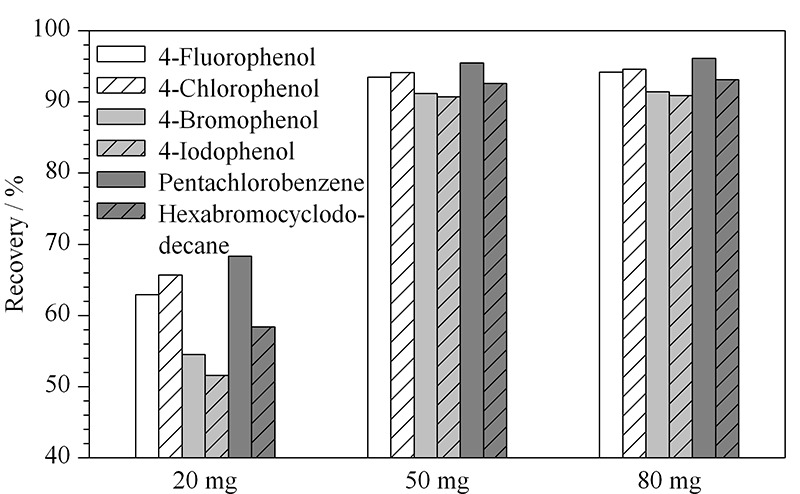
活性炭用量对样品加标回收率的影响

2.1.3 燃烧气及其流量的选择

对于活性炭的燃烧,首选燃烧气应是氧气,考虑到燃烧气的杂质对检测背景的影响,纯度应不小于99.9%。对于有机卤素燃烧炉来说,内管和外管均通氧气能够确保燃烧处于富氧条件,确保样品燃烧过程的完全转化。对于燃烧气流量的选择,原则上应选择较大流量,以确保样品的充分燃烧,但过大的流量一方面是浪费,另一方面对吸收液冲击太大,而且燃烧过程产生的烟雾易被吹散到燃烧管出口端,易形成过多残留从而使得测定结果偏低。通过多次试验比较,选择内管氧气流量为100~120 mL/min,外管氧气流量为40~60 mL/min。

2.1.4 燃烧升温程序的选择

对于吸附AOX的活性炭来说,它们在高温氧化燃烧过程中易发生一些化学变化,大分子物质首先在低温下进行裂解,之后在高温富氧条件下进行燃烧,其反应是逐步进行的,根据这种燃烧特点,设置程序升温的方式能够让燃烧彻底进行。前处理的最后一步过程是抽滤,得到的活性炭和滤膜含有少量水分,因此需要设置初始温度100 ℃保持2 min让水分蒸发后再逐步升温进行燃烧。通过多次比较,采用100 ℃保持2 min,之后15 min内升至950 ℃保持5 min,继续通入氧气进行吹脱5 min的程序升温方式能够使得燃烧产生的气体尽可能被吸收完全,从而获得好的检测结果。

2.1.5 吸收方式及级数的选择

本方法采用多孔气体吸收瓶进行吸收,这样能够使气体分散为细小气泡,增加气体与吸收液的接触面积,从而提高吸收率;此外,还可使用多个多孔气体吸收瓶串联进行二级、三级吸收,以进一步提高气体的吸收率。选取阴性加标样品(50 mg/kg)分别在一级吸收、二级吸收和三级吸收的条件下进行回收试验,结果见图3。从图中可以看出,第二级吸收中仍含少量残余卤素,第三级中几乎不含有卤素,因此本方法采用二级吸收。

**图 3:**
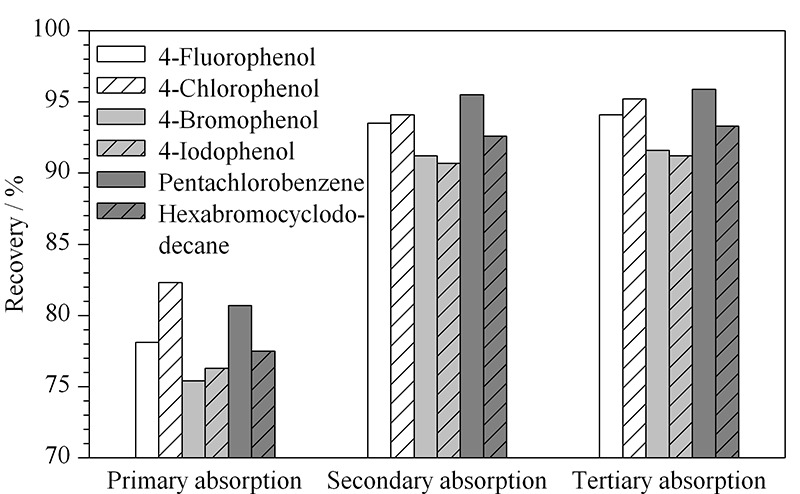
吸收级数对样品加标回收率的影响

2.1.6 吸收液的选择

按照理论,样品中的AOX完全燃烧产生的卤化氢气体能够被碱性溶液吸收,因此需要选择合适的吸收液。一是强碱性溶液比如氢氧化钠溶液,它对各种酸性气体有很好的吸收,但其碱性太强会使离子色谱中目标物的峰形变差;二是弱碱性溶液比如离子色谱淋洗液,三是纯水。此外,有文献^[21,22]^提到在吸收液中加入0.3%~3%的过氧化氢以提高对卤素的吸收,但它的强氧化性对色谱柱内填料的损伤较大,且国内购置的过氧化氢含有氟、氯等杂质,会对痕量卤素的检测带来干扰,所以本方法不添加过氧化氢。

针对上述问题,分别选取离子色谱淋洗液和超纯水作为吸收液进行阴性加标样品(50 mg/kg)的回收试验,结果见图4。从图中可以看出,离子色谱淋洗液作为吸收液时,目标物的回收率较高,且色谱图中干扰峰较少,有利于测定。

**图 4:**
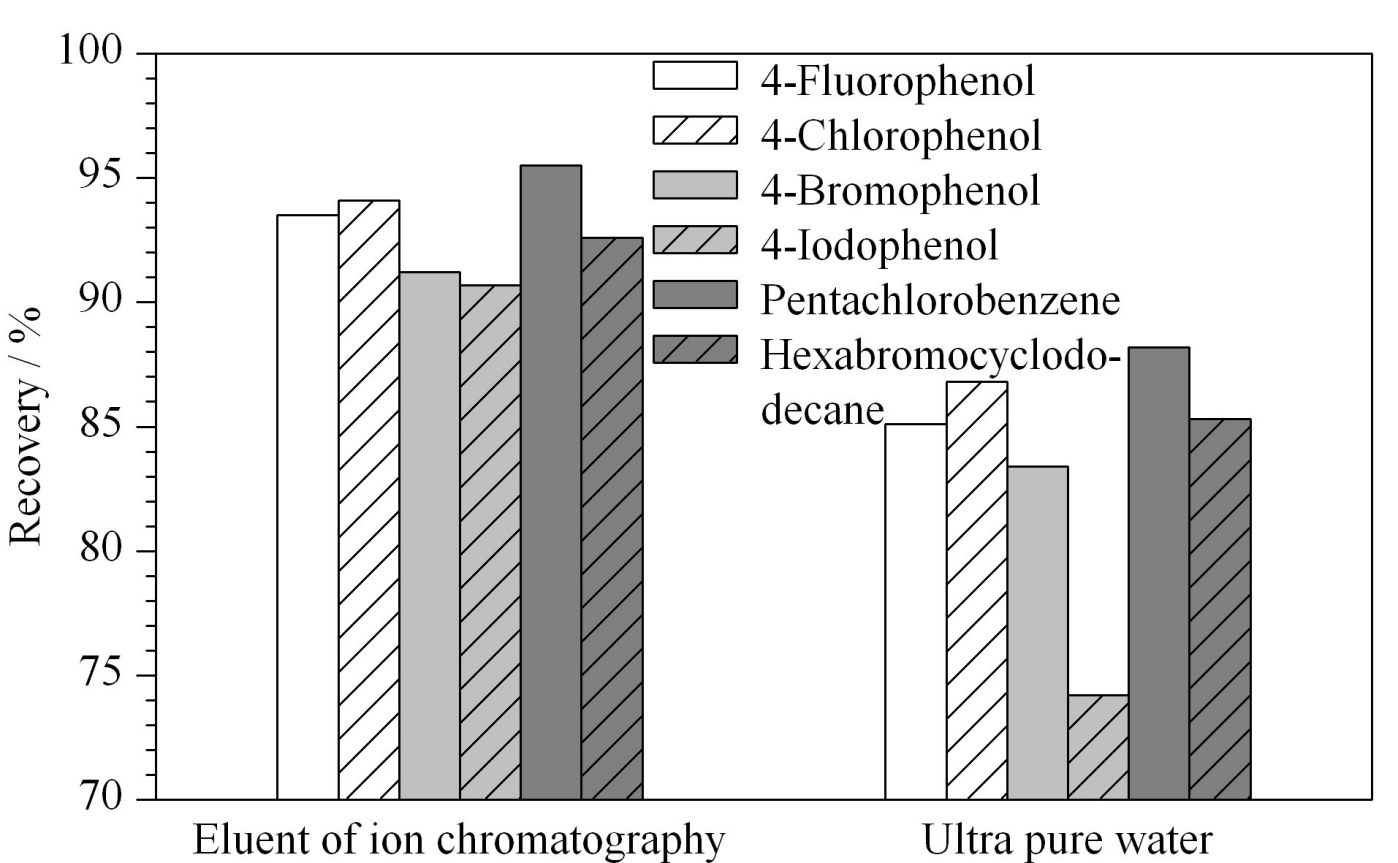
不同吸收液对样品加标回收率的影响

2.1.7 空白试验

为了考察所用试剂、材料和试验环境等条件对测定结果的影响,在不添加样品的情况下按照与样品测定相同步骤做全程序空白试验,结果发现空白中会有微量AOF、AOCl的存在,因此在样品测试时需同时做空白试验,以减少干扰。

### 2.2 离子色谱条件的选择

色谱柱是色谱分离技术的最核心部分,根据离子色谱仪厂商提供的色谱柱适用条件,针对F^-^、Cl^-^、Br^-^、I^-^测定的色谱柱可选择Metrosep A Supp 5-250/4.0型阴离子分析柱,它是一种碳酸盐体系的阴离子交换柱,其固定相填料为含季铵盐基团的聚乙烯醇,粒径为5 μm,柱容量为107 μmol(Cl^-^),这为F^-^、Cl^-^、Br^-^、I^-^等组分与基体的分离提供了先决条件。因此,通过选择合适的色谱条件,能够在40 min内对F^-^、Cl^-^、Br^-^、I^-^进行分离,具有检测灵敏度高、重复性好、数据准确度高等优势,样品中可能存在的高浓度硝酸根、硫酸根、碳酸根和磷酸根均不会对卤素测试产生干扰。

Metrosep A Supp 5-250/4.0型色谱柱适用温度范围为20~60 ℃,流速不超过0.8 mL/min。通过试验发现,随着色谱柱温度及流速的增加,F^-^、Cl^-^、Br^-^、I^-^的色谱峰保留时间不断缩短,但是在纺织样品测定中发现,Br^-^附近有干扰峰存在,通过调节不同的温度及流速,选择35 ℃和0.7 mL/min作为检测最佳条件,能够确保Br^-^不受干扰峰的影响。在选定的离子色谱条件下,F^-^、Cl^-^、Br^-^、I^-^的保留时间和典型离子色谱图见图5。

**图 5:**
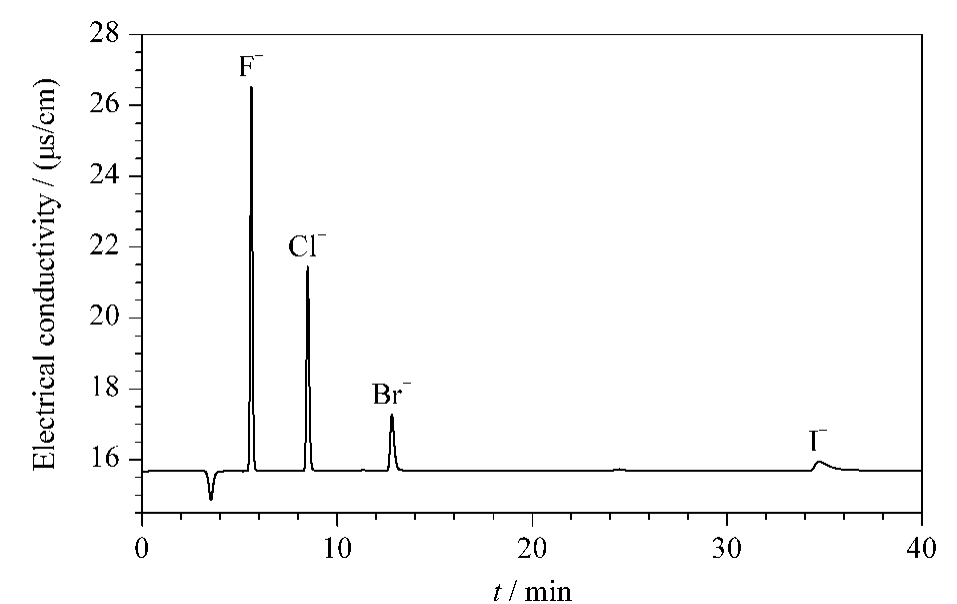
F^-^、Cl^-^、Br^-^、I^-^的典型离子色谱图

### 2.3 方法学评价

2.3.1 线性范围、方法检出限和定量限

在本方法所确定的实验条件下,对配制的一系列不同浓度的氟、氯、溴、碘离子的混合标准工作溶液进行测定,其质量浓度与响应值有良好的线性关系,使用线性最小二乘法以色谱峰面积(*Y*)对卤素离子的质量浓度(*X*, mg/L)进行线性拟和,得到标准曲线的回归方程,线性相关系数(*R*^2^)均大于0.999,表明4种卤素离子在0.02~10 mg/L范围内线性关系良好。

在基质匹配的标准溶液中,根据3倍和10倍信噪比分别确定卤素离子的仪器检出限和定量限,同时根据样品前处理的定容体积25 mL、试样量2 g计算得到方法的检出限(*S/N*=3)和定量限(*S/N*=10),结果详见表1。

**表 1:** 卤素离子的保留时间、回归方程、相关系数、检出限和定量限

Halogen ion	Retention time/min	Regression equation	R^2^	LOD/(mg/kg)	LOQ/(mg/kg)
F^-^	5.60	Y=1.51×10^-2^X-2.07×10^-2^	0.9997	0.020	0.066
Cl^-^	8.49	Y=9.83×10^-3^X-2.24×10^-2^	0.9995	0.018	0.059
Br^-^	12.81	Y=3.88×10^-3^X-3.36×10^-3^	0.9999	0.038	0.125
I^-^	34.71	Y=2.24×10^-3^X-2.14×10^-3^	0.9999	0.088	0.250

*Y*: peak area; *X*: mass concentration, mg/L.

考虑到国内外技术法规和标准对纺织品中AOX的限量要求及方法的普适性,将本方法的定量限定义为:AOF 0.10 mg/kg、AOCl 0.10 mg/kg、AOBr 0.25 mg/kg、AOI 0.50 mg/kg。

2.3.2 回收率和精密度

以棉、毛和涤纶3种不同种类的阴性纺织样品作为研究对象,选取典型的水溶性有机卤化物(4-氟苯酚、4-氯苯酚、4-溴苯酚和4-碘苯酚)和脂溶性有机卤化物(五氯苯和六溴环十二烷)进行样品中AOX的回收率和精密度试验。分别在添加卤素各组分水平为5、50和125 mg/kg时进行前处理及燃烧试验,同时做空白试验,用外标法进行定量,每个水平单独测定7次,扣除空白后的平均回收率和相对标准偏差(RSD)详见表2。

**表 2:** 阴性样品中AOX的平均加标回收率和相对标准偏差(*n*=7)

Organic halide	Added^*^/ (mg/kg)	Cotton			Wool			Polyester fiber	
		Recovery/%	RSD/%		Recovery/%	RSD/%		Recovery/%	RSD/%
4-Fluorophenol	5	88.6	3.6		89.0	3.0		88.3	2.8
	50	90.7	3.3		90.8	3.2		90.4	4.5
	125	95.7	2.2		95.3	2.0		95.8	2.1
4-Chlorophenol	5	90.9	5.3		92.1	4.0		89.9	2.5
	50	91.6	3.5		92.8	3.1		91.7	3.4
	125	98.1	3.1		98.7	2.8		95.9	2.1
4-Bromophenol	5	85.9	5.5		85.1	4.1		85.1	5.7
	50	87.4	4.8		86.7	4.0		86.5	4.1
	125	93.4	2.4		92.6	2.2		93.6	2.6
4-Iodophenol	5	83.7	5.2		84.7	4.3		82.3	4.2
	50	85.7	4.9		85.4	4.3		85.5	3.2
	125	91.2	2.9		92.4	2.9		91.7	2.7
Pentachlorobenzene	5	85.7	3.2		86.0	4.9		87.3	3.7
	50	86.5	2.8		87.7	4.4		89.4	4.4
	125	93.4	3.1		92.8	3.9		92.5	3.3
Hexabromocyclododecane	5	84.6	5.2		86.0	4.0		85.3	4.4
	50	87.1	4.0		86.0	5.3		85.6	3.9
	125	89.6	3.0		90.3	2.4		90.2	4.0

* Converted to halogen content.

从表2的数据可以看出,AOX在棉、毛和涤纶阴性样品中测得加标的平均回收率为82.3%~98.7%,相对标准偏差为2.0%~5.7%(*n*=7),说明该方法对水溶性有机卤化物和脂溶性有机卤化物的加标回收率均能满足要求,表明本方法准确、可靠。

### 2.4 实际样品分析

应用本文建立的方法对多个纺织样品进行测定,同时做空白试验,扣除空白后的检测结果显示在蓝色涂层涤纶面料、黑色莫代尔面料两个样品中检出了不同含量的AOX,详见表3。

**表 3:** 实际样品的测定结果

AOX	Blue coated polyester fabric/(mg/kg)				Black modal fabric/ (mg/kg)		
	1	2	3		1	2	3
AOF	14.5	13.8	14.9		0	0	0
AOCl	26.9	27.8	25.4		8.2	8.6	7.9
AOBr	0	0	0		3.3	3.1	3.4
AOI	0	0	0		0	0	0

AOF: adsorbable organic fluorine; AOCl: adsorbable organic chlorine; AOBr: adsorbable organic bromine; AOI: adsorbable organic iodine.

从结果可以看出,样品中各检出组分3次测定的RSD为3.9%~4.7%,表明该方法的重复性较好,适合纺织品中AOX的测定。其中典型样品蓝色涂层涤纶面料的离子色谱图见图6。

**图 6:**
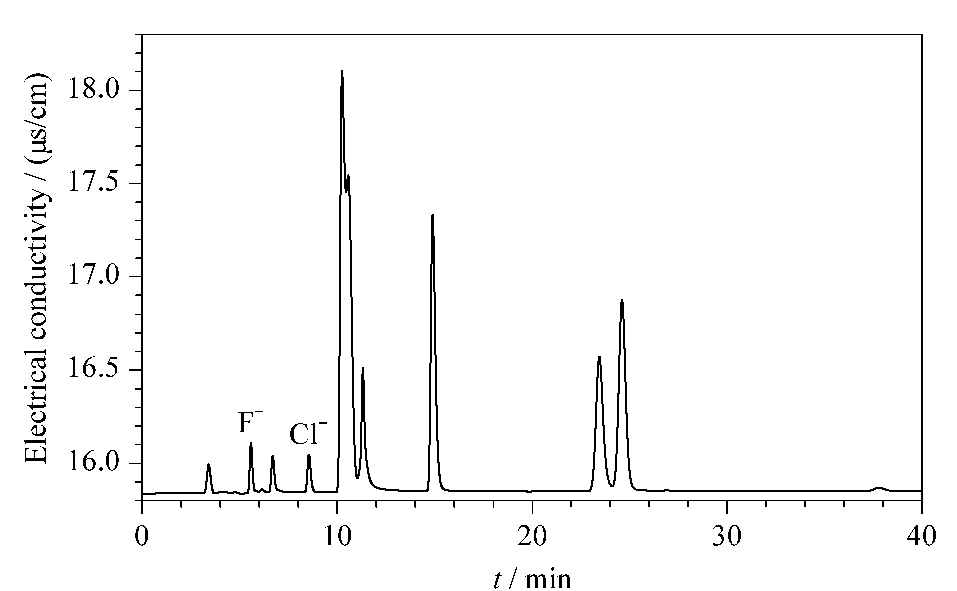
典型样品蓝色涂层涤纶面料的离子色谱图

## 3 结论

本文建立了超声提取-高温燃烧吸收-离子色谱同时测定纺织品中AOF、AOCl、AOBr和AOI的检测新方法。该方法通过采用活性炭的振荡吸附、程序升温的高温氧化燃烧方式和多孔吸收瓶的二级吸收方法,能够将AOX彻底转化为无机卤素并被充分吸收形成阴离子;同时利用离子色谱仪选择性好、灵敏度高的特点成功地一次性分离检测4种AOX,且无杂质离子的干扰。本方法简单、准确、可靠,满足国内外技术法规和标准对纺织品中AOX的限量要求,适用于纺织品中AOX的检测分析。
